# Composition-Driven Structural Evolution in Cobalt–Manganese Glycerates toward Defect-Rich Amorphous Electrodes for High-Performance Battery–Supercapacitor Hybrids

**DOI:** 10.1021/acsomega.6c07626

**Published:** 2026-08-10

**Authors:** Tsai-Mu Cheng, Chih-Yu Chang, Zher-Yu You, Chutima Kongvarhodom, Sadang Husain, Sibidou Yougbaré, Hung-Ming Chen, Lu-Yin Lin

**Affiliations:** † Graduate Institute for Translational Medicine, College of Medical Science and Technology, Taipei Medical University, Taipei 11031, Taiwan; ‡ Taipei Heart Institute, Taipei Medical University, Taipei 11031, Taiwan; § Cardiovascular Research Center, Taipei Medical University Hospital, Taipei 11031, Taiwan; ∥ Department of Chemical Engineering and Biotechnology, 34877 National Taipei University of Technology, Taipei 106344, Taiwan; ⊥ Department of Chemical Engineering, King Mongkut’s University of Technology Thonburi, 126 Pracha-u-thit, Toong-kru, Bangkok 10140, Thailand; # Department of Physics, Faculty of Mathematics and Natural Science, 175513 Lambung Mangkurat University, Banjarmasin 70124, Indonesia; ∇ Institut de Recherche en Sciences de la Santé (IRSS-DRCO)/Nanoro, 03 B.P 7192 Ouagadougou 03, Burkina Faso; ○ Gingen Technology Co., LTD., Rm. 7, 10F., No.189, Sec. 2, Keelung Rd., Xinyi Dist., Taipei 11054, Taiwan

## Abstract

The development of electrode materials with efficient charge transfer and abundant active sites remains crucial for high-performance supercapacitors. In this work, cobalt–manganese glycerates with tunable cobalt-to-manganese ratios are synthesized to systematically investigate the relationship between composition, structure, and electrochemical behavior. The incorporation of manganese disrupts the regular growth of cobalt-based glycerates, resulting in amorphous, rough, and defect-rich structures with enhanced surface area and improved electrolyte accessibility. Among all compositions, the optimized bimetallic sample with the highest manganese content exhibits the highest specific capacitance of 1333.6 F/g at 20 mV/s, demonstrating a balanced combination of electrical conductivity and redox activity. Compared with monometallic counterparts, the bimetallic system shows improved charge storage capability due to synergistic electronic interaction and more efficient ion and electron transport. The assembled two-electrode device achieves a potential window of 1.3 V and a maximum energy density of 83 Wh/kg at 325 W/kg. In addition, the device retains 90% of its initial capacitance with nearly 100% Coulombic efficiency after 7500 cycles. These results highlight the importance of composition regulation in glycerate-derived materials, and further improvements may be achieved by optimizing composition and enhancing structural stability.

## Graphical abstract

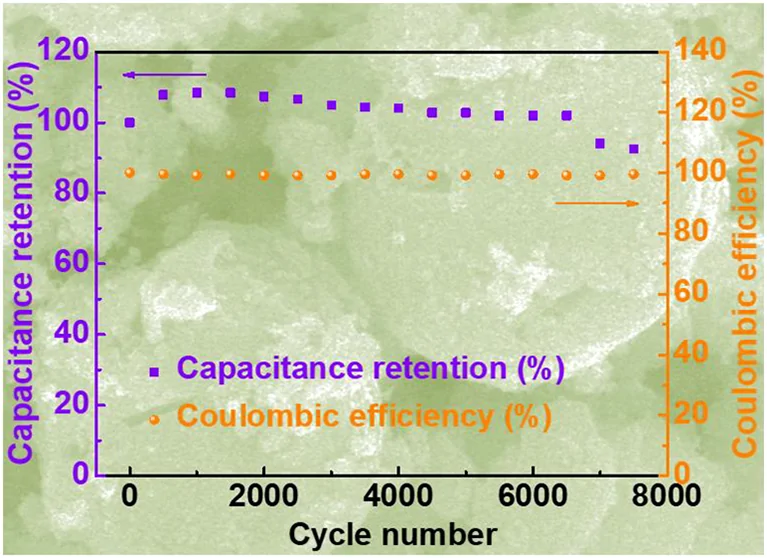

## 1 Introduction

Supercapacitors can generally be classified into electric double-layer capacitors (EDLCs) and pseudocapacitors according to their charge-storage mechanisms. EDLCs store energy through electrostatic ion adsorption at the electrode/electrolyte interface, providing excellent cycling stability and high power density but relatively limited energy density. In contrast, pseudocapacitors rely on fast and reversible Faradaic redox reactions occurring on or near the electrode surface, offering significantly higher charge-storage capability. Consequently, recent research has increasingly focused on designing transition-metal-based electrode materials with abundant redox-active sites, defect-rich structures, and optimized electronic configurations to simultaneously improve capacitance, rate capability, and cycling stability. Composition engineering has recently emerged as an effective strategy for regulating the local coordination environment, electronic structure, and charge-transfer kinetics, thereby enhancing the electrochemical performance of pseudocapacitive materials.^1–3^ These advances have attracted considerable attention for developing next-generation high-performance supercapacitors.^4–6^ On the other hand, the increasing demand for high-performance energy storage systems has driven the development of battery–supercapacitor hybrids (BSHs), which aim to integrate the high energy density of batteries with the high power density and long cycle life of supercapacitors.^7–9^ In such systems, the properties of electrode materials are critical, as they directly determine charge storage mechanisms, rate capability, and overall device performance.^10–12^

Metal glycerates have recently attracted increasing attention as promising precursors and active materials for electrochemical energy storage.^13–15^ Their three-dimensional coordination frameworks, constructed through metal–oxygen–carbon linkages, offer structural flexibility and abundant redox-accessible sites.^16^ In addition, the polyol environment provides intrinsic carbon sources and facilitates uniform dispersion of metal centers, which is beneficial for enhancing electrical conductivity after subsequent treatments. More importantly, the synthesis of metal glycerates allows multiple metal ions to be incorporated into a single framework under relatively mild conditions, enabling precise compositional control at the molecular level.^17,18^ These characteristics make glycerate systems an ideal platform for probing composition–structure–property relationships in multimetallic architectures. Transition metal-based systems, particularly those involving multiple redox-active species, provide an effective route to further enhance the performance of glycerate-derived materials. Cobalt-based compounds are known for their fast Faradaic reaction kinetics and relatively good electrical conductivity, enabling rapid charge transfer processes.^19–21^ Kubendhiran et al. synthesized honeycomb-like cobalt-nickel-molybdenum layered double hydroxides (CoNiMo-LDHs) via a magnetic stirring method, utilizing NiCo-glycerate spheres prepared through a solvothermal process as sacrificial templates. The CoNiMo-LDH exhibits a specific capacitance of 1059 F/g at 3 A/g.^13^ In contrast, manganese-based materials offer multiple accessible oxidation states, low cost, and environmental compatibility, making them attractive for large-scale applications.^22–25^ Wei et al. presented a glycerate template strategy to synthesize Ni–Mn–Ce oxide hierarchical hollow architectures via solvothermal treatment as a supercapacitor electrode material. The Ni–Mn–Ce oxides achieved a specific capacity of 688 C/g at 2.0 A/g.^26^ However, each single-metal system also presents inherent limitations. Cobalt-based materials may suffer from insufficient structural stability during prolonged cycling, whereas manganese-based materials often exhibit limited electrical conductivity, restricting their rate performance. The integration of two metal species into a bimetallic system provides an effective pathway to overcome these drawbacks. Through electronic interaction between different metal centers, bimetallic materials can achieve synergistic effects, including enhanced redox activity, improved charge transfer efficiency, and optimized active site distribution.^27–31^ Importantly, such synergy is highly sensitive to the relative proportion of each component. Variations in precursor ratios can alter nucleation processes, coordination environments, and local electronic structures, thereby significantly affecting both morphology and electrochemical behavior. Teng et al. used a metal-glycerate precursor strategy to synthesize MnCo_2_O_4_ hollow spheres for an all-solid-state asymmetric microsupercapacitor.^32^ Despite these advantages, studies on glycerate-based systems have primarily focused on morphology regulation^33^ or derivative formation,^34,35^ while the fundamental role of compositional variation remains largely unexplored. In particular, the correlation between precursor ratio, coordination environment, and electrochemical behavior has not been systematically clarified. Therefore, a comprehensive investigation that links precursor composition with structural evolution and charge storage characteristics in bimetallic glycerate systems is highly desirable. Therefore, the Co–Mn combination was selected as a representative bimetallic system to investigate how compositional regulation influences the coordination structure, defect formation, and electrochemical performance.

In this work, cobalt–manganese glycerates with controlled precursor ratios are synthesized via a solvothermal coordination approach. By systematically tuning the Co/Mn ratio, the relationship between composition, structural evolution, and electrochemical performance is elucidated. Monometallic counterparts are also prepared for comparison to clarify the contribution of bimetallic interactions. Unlike previous studies that mainly focus on morphology engineering or the conversion of glycerates into derivative materials such as oxides and sulfides, this work directly investigates the intrinsic electrochemical behavior of bimetallic glycerates through systematic composition regulation. By eliminating the influence of postsynthetic phase transformation, a clear composition–structure–property relationship is established, providing fundamental insights for the rational design of glycerate-based and glycerate-derived electrode materials. Although metal glycerates are commonly employed as sacrificial precursors for synthesizing oxides, sulfides, phosphides, and other derivative materials, the conversion process often introduces additional variables, including phase transformation, crystallization, and carbonization, making it difficult to isolate the intrinsic effect of precursor composition. Therefore, this work deliberately focuses on glycerates themselves to establish a direct composition–structure–property relationship. The knowledge obtained from this study provides a fundamental guideline for the rational design of glycerate-derived functional materials in future investigations.

## 2 Experimental Section

### 2.1 Preparation of Bimetallic and Monometallic Glycerates

Bimetallic cobalt–manganese glycerates (CoMnG) were first synthesized with varying precursor ratios to investigate the compositional effect on the material properties. In a typical procedure, cobalt nitrate hexahydrate (Co­(NO_3_)_2_·6H_2_O, Acros, 98%) and manganese nitrate hexahydrate (Mn­(NO_3_)_2_·6H_2_O, thermos scientific, 98%) with different molar ratios (total metal amount fixed at 1 mmol) were codissolved in a mixed solvent containing 10 mL of glycerol (Echo, 99.5%) and 70 mL of isopropanol (Echo, 99.5%). The solution was ultrasonicated for 10 min to form a homogeneous mixture, followed by solvothermal treatment at 120 °C for 5 h in a Teflon-lined autoclave to facilitate coordination-driven nucleation and growth. The hydrothermal temperature of 120 °C was selected based on our preliminary experiments, which confirmed that this temperature could reproducibly produce homogeneous glycerate products with good morphology. After naturally cooling to room temperature, the precipitates were collected by centrifugation, thoroughly washed with deionized water and ethanol, and dried at 60 °C for 4 h. The CoMnG synthesized using the Co:Mn ratio of 2:8, 4:6, 5:5, 6:4 and 8:2 is nominated as Co2Mn8G, Co4Mn6G, Co5Mn5G, Co6Mn4G and Co8Mn2G, respectively.

For comparison, monometallic glycerates were also prepared under identical conditions using 0.5 mmol of a single metal precursor. Specifically, cobalt glycerate (CoG) and manganese glycerate (MnG) were synthesized by respectively dissolving 0.5 mmol of Co­(NO_3_)_2_·6H_2_O or Mn­(NO_3_)_2_·6H_2_O in the same solvent system, followed by the same solvothermal and post-treatment processes. These monometallic samples were used as references to elucidate the synergistic effects in bimetallic systems. The illustration of the experimental processes for synthesizing CoG, MnG, and CoMnG is given in Scheme 1.

**Scheme 1:**
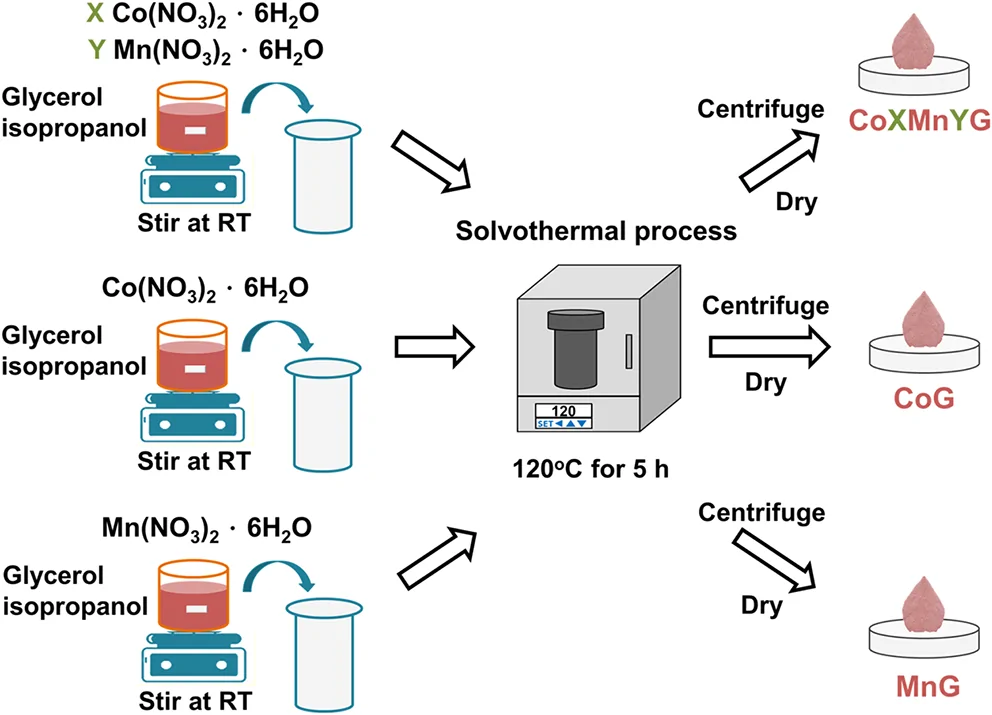
Illustration of the Experimental Processes for Synthesizing CoMnG, CoG, and MnG

### 2.2 Electrode Preparation and Device Assembly

Electrodes were constructed using nickel foam as a conductive substrate. Prior to coating, the substrate was treated to eliminate surface contaminants and improve interfacial contact. The active material was blended with conductive carbon and poly­(tetrafluoroethylene) (PTFE) binder at a weight ratio of 7:2:1.^36^ The mixture was then dispersed in a solution containing equal volumes of ethanol and deionized water to form a slurry, followed by continuous agitation for 24 h to ensure uniform dispersion. The resulting paste was applied onto the nickel foam and subsequently dried at 60 °C to remove residual solvent. The amount of active material deposited was carefully controlled to maintain consistency across samples. For practical evaluation, an asymmetric supercapacitor was assembled. The optimized CoMn–G electrode was employed as the positive electrode, while reduced graphene oxide (rGO) was used as the negative electrode. An aqueous KOH solution (3 M) served as the electrolyte. The mass ratio between the two electrodes was adjusted according to the charge balance requirement. The mass ratio between the two electrodes was adjusted according to the charge balance requirement. The mass ratio between the positive and negative electrodes was determined according to the charge balance equation as follows (eq 1).^36–38^

1 $$C_{+}\times \Delta V_{+}\times m_{-}=C_{-}\times \Delta V_{-}\times m_{+}$$

where *C*_+_ and *C*_–_ are the specific capacitances of the positive and negative electrodes, Δ*V*_+_ and Δ*V*_–_ are the potential windows of the positive and negative electrodes, and *m*_–_ and *m*_+_ are the loading masses of the positive and negative electrodes, respectively.

### 2.3 Material and Electrochemical Characterizations

The morphology of the synthesized materials was examined using field-emission scanning electron microscopy (FE-SEM, Nova NanoSEM 230, FEI, USA). Crystalline structures were analyzed by X-ray diffraction (XRD, X’Pert^3^ Powder, PANalytical). The surface chemical composition and valence states were further characterized by X-ray photoelectron spectroscopy (XPS, VG Scientific ESCALAB 250) with Al Kα radiation. Electrochemical properties were evaluated in a standard three-electrode configuration, employing a platinum foil as the counter electrode and an Ag/AgCl electrode as the reference. Cyclic voltammetry (CV), galvanostatic charge–discharge (GCD), and electrochemical impedance spectroscopy (EIS) measurements were performed using an Autolab PGSTAT204 electrochemical workstation equipped with an FRA2 module. The impedance spectra were collected over a frequency range from 0.01 Hz to 100 kHz under open-circuit conditions. Three samples for each condition are tested to confirm reproducibility.

## 3 Results and Discussion

### 3.1 Material Analysis of CoMnG Synthesized Using Different Metal Precursor Ratios

Figure 1 shows the SEM images of CoMnG samples synthesized with different Co/Mn precursor ratios. A clear evolution in morphology is observed as the composition varies, indicating that the relative ratio of Co to Mn plays a decisive role in regulating the nucleation and growth behavior of the glycerate structures.

**Figure 1:**
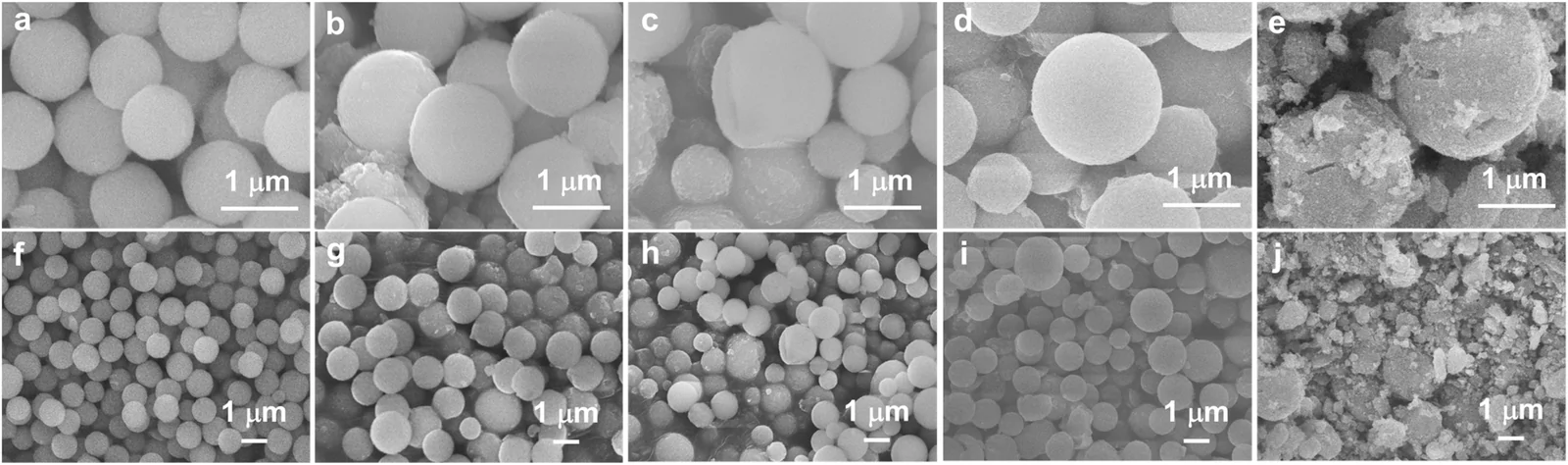
SEM images of (a, f) Co8Mn2G, (b, g) Co6Mn4G, (c, h) Co5Mn5G, (d, i) Co4Mn6G, and (e, j) Co2Mn8G.

At a high Co content, i.e., Co8Mn2G in Figure 1a,f, the sample exhibits well-defined and uniformly distributed microspheres with relatively smooth surfaces, suggesting a controlled growth process dominated by Co-centered coordination. As the Mn content increases, i.e., Co6Mn4G in Figure 1b,g and Co5Mn5G in Figure 1c,h, the spherical morphology is largely retained, but the particle size distribution becomes broader, and the surfaces become noticeably rougher. In addition, the particle size gradually increases with increasing Mn content, suggesting that Mn incorporation perturbs the growth kinetics, thereby promoting particle coarsening, increased surface irregularity, and partial structural heterogeneity. With further increase in Mn content, i.e., Co4Mn6G in Figure 1d,i, the uniformity of the microspheres is further reduced, accompanied by the appearance of smaller particles and partial aggregation. Although the spherical morphology is no longer well-maintained, the particle surfaces exhibit abundant protrusions and rough features. At the highest Mn ratio, i.e., Co2Mn8G in Figure 1e,j, the spherical morphology is almost completely disrupted, and the sample is dominated by irregular agglomerates composed of fine nanoparticles. Notably, these structures are decorated with numerous nanoscale protrusions that can significantly increase the specific surface area and provide more active sites for electrochemical reactions.

The observed composition-dependent morphology can be attributed to the distinct coordination characteristics of Co^2+^ and Mn^2+^ ions in the glycerol-based solvent system. Co^2+^ ions tend to form more stable coordination complexes with glycerol, which promotes isotropic growth and the formation of well-defined microspheres. In contrast, Mn^2+^ ions exhibit relatively weaker coordination interactions, leading to faster nucleation and less controlled aggregation. Meanwhile, the accelerated nucleation and subsequent particle coalescence in Mn-rich systems may contribute to the formation of larger particle sizes and roughened surfaces. Therefore, increasing Mn content shifts the balance from growth-dominated to nucleation-dominated processes, resulting in progressively disordered structures. Such structural evolution is expected to significantly influence the electrochemical properties. The well-defined spherical structures at higher Co ratios may provide efficient electron transport pathways and structural stability, whereas the increased surface roughness and reduced particle size at moderate Mn contents could enhance the accessible active surface area. These protrusions are advantageous for enhancing electrode–electrolyte contact and increasing the accessible active surface area. However, excessive Mn content may lead to structural collapse and aggregation, which could hinder ion diffusion and reduce effective utilization of active sites. These results suggest that an optimal Co/Mn ratio is critical for balancing structural integrity and electrochemical activity.

The XRD patterns of CoMnG samples with different Co/Mn ratios are shown in Figure 2a. All samples exhibit broad and featureless diffraction profiles over the 2θ range of 20–80°, indicating the predominantly amorphous nature of the synthesized bimetallic glycerates. No characteristic diffraction peaks of crystalline cobalt- or manganese-based phases are observed, confirming the absence of phase segregation. Notably, the structural features show a composition-dependent evolution. The intermediate compositions (Co4Mn6G, Co5Mn5G, and Co6Mn4G) display relatively smooth and uniform scattering profiles, suggesting a more homogeneous amorphous structure. In contrast, the Co-rich (Co8Mn2G) and Mn-rich (Co2Mn8G) samples exhibit more pronounced intensity fluctuations and broader diffuse features, indicating increased structural disorder and heterogeneity. This behavior suggests that a balanced Co/Mn ratio facilitates more uniform coordination and network formation during the solvothermal process, whereas excessive dominance of either metal species disrupts the growth equilibrium, leading to structural irregularity and heterogeneity. Unlike previously reported crystalline metal glycerolates that exhibit a characteristic diffraction peak at approximately 2θ = 9–10°, no distinct diffraction peak was observed in the present samples. Instead, a broad and featureless diffraction pattern was obtained throughout the low-angle region, indicating the predominantly amorphous nature of the synthesized Co–Mn glycerolates. The absence of the characteristic low-angle reflection is attributed to the low crystallinity and structural disorder introduced by the bimetallic coordination environment. This interpretation is further supported by the FTIR spectra, which confirm the coordination between glycerol ligands and Co/Mn ions, thereby verifying the successful formation of the metal glycerolate framework despite its amorphous structure.

**Figure 2:**
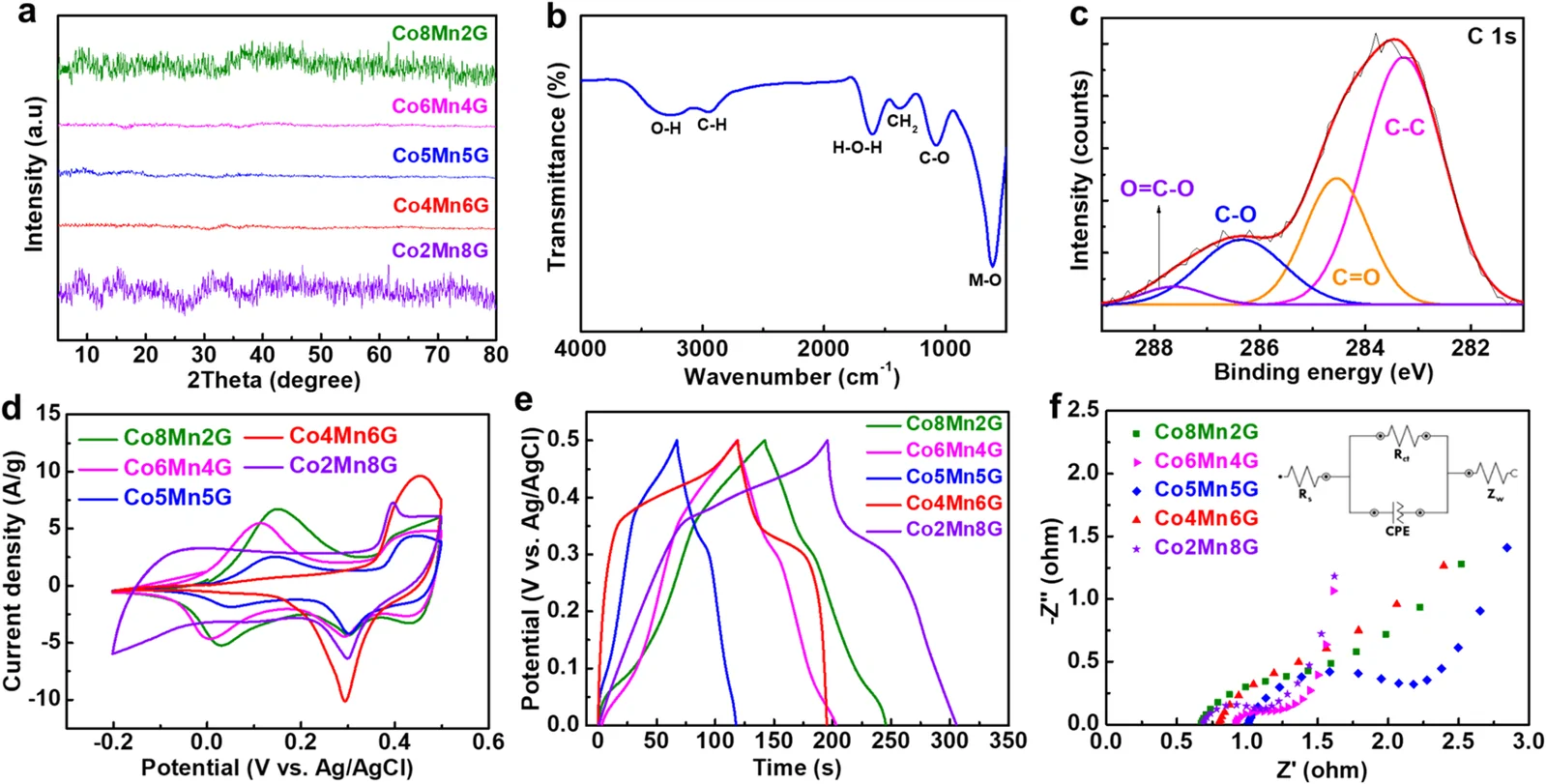
(a) XRD patterns, (b) FTIR spectrum (M–O = metal-to-oxygen bond), (c) C 1s spectrum, (d) CV curves, (e) GCD curves, (f) Nyquist plots with the corresponding equivalent circuit of CoMnG.

Figure 2b presents the FTIR spectra of the synthesized Co–Mn glycerolates. A broad absorption band centered at approximately 3200–3500 cm^–1^ is assigned to the stretching vibration of hydroxyl (−OH) groups from coordinated glycerol and adsorbed water. The bands located near 2920 and 2850 cm^–1^ correspond to the asymmetric and symmetric stretching vibrations of aliphatic C–H bonds. The strong absorption band around 1050–1120 cm^–1^ is attributed to the C–O stretching vibration of glycerate ligands. Furthermore, characteristic bands appearing in the 500–700 cm^–1^ region are assigned to metal–oxygen (M–O) coordination vibrations, indicating that glycerol molecules are chemically coordinated with Co and Mn ions to form the metal glycerate framework. These FTIR results further support the successful synthesis of Co–Mn glycerolates. The high-resolution C 1s spectrum (Figure 2c) can also confirm the successful synthesis of glycerolates. This spectrum can be deconvoluted into four components corresponding to C–C, C=O, C–O, and O–C=O species, respectively. The presence of the O–C=O component is consistent with the coordinated glycerate ligand and agrees well with the FTIR results, confirming the successful formation of the metal glycerolate framework.

The electrochemical properties of CoMnG electrodes with different Co/Mn ratios were systematically investigated by CV, GCD, and Nyquist plots, as shown in Figure 2d–f. The CV curves in Figure 2d exhibit pronounced redox peaks, confirming that the charge storage mechanism is dominated by Faradaic reactions associated with reversible Co^2+^/Co^3+^ and Mn^2+^/Mn^3+^ redox couples. Specifically, two distinct pairs of redox peaks are observed in the CV curves of Co8Mn2G, Co4Mn6G, Co5Mn5G, and Co6Mn4G electrodes. The anodic peaks are located at approximately 0.1–0.2 and 0.4–0.5 V vs Ag/AgCl, while the corresponding cathodic peaks appear at around 0.0–0.1 and 0.3–0.35 V vs Ag/AgCl. These redox couples are attributed to the reversible oxidation and reduction processes of Co and Mn species in the alkaline electrolyte. These peaks can be generally described by the redox reactions as follows. Co­(OH)_2_ + OH^–^ ↔ CoOOH + H_2_O + e^–^ and Mn­(OH)_2_ + OH^–^ ⇌ MnOOH + H_2_O + e^–^. In contrast, the CV curve of the Co2Mn8G electrode exhibits a quasi-rectangular shape in the low-potential region, along with only one pronounced pair of redox peaks in the higher potential region (>0.3 V vs Ag/AgCl). This behavior indicates a shift toward dominant pseudocapacitive characteristics. The high Mn content in Co2Mn8G favors fast surface redox reactions and capacitive charge storage, thereby suppressing well-defined Faradaic peak features at lower potentials that are typically associated with Co-based redox processes. Moreover, the specific capacitance and specific capacity based on CV measurements are determined from the integrated area of the voltammogram according to eqs 2 and 3 as follows.^20,39,40^

2 $$C_{F}=\frac{\int IdV}{\Delta V\cdot \nu }$$

3 $$\mathrm{specific}\;\mathrm{capacity}=C_{F}\times \Delta V=I\times \Delta t$$

where ∫*I*d*V* represents the integrated area under the CV curve, and ν is the scan rate (V/s). The integrated area of CV curves shows a clear dependence on composition, where Co2Mn8G and Co8Mn2G exhibit significantly larger enclosed areas compared to intermediate compositions. Table 1 shows the specific capacitance and specific capacity of CoMnG calculated using the CV curves at 20 mV/s. Quantitatively, the Co2Mn8G electrode delivers the highest specific capacitance of 1333.6 F/g, corresponding to the specific capacity of 933.5 C/g, followed by Co8Mn2G (1268.7 F/g and 888.1 C/g), while Co5Mn5G shows the lowest values (649.7 F/g and 454.8 C/g). The superior electrochemical performance of Co2Mn8G can be primarily attributed to its unique structural features. As observed in SEM images, Co2Mn8G exhibits highly irregular agglomerates composed of fine nanoparticles decorated with abundant nanoscale protrusions, which significantly increase the specific surface area and expose a large number of electrochemically active sites. This morphology facilitates enhanced electrode–electrolyte contact and promotes fast surface redox reactions. In addition, XRD results indicate a more disordered amorphous structure for Co2Mn8G, which is beneficial for ion diffusion and provides more accessible pathways for electrolyte penetration. From a theoretical perspective, Mn-rich systems tend to favor pseudocapacitive behavior with fast and reversible surface reactions, thereby contributing significantly to the overall capacitance. In contrast, the inferior performance of Co5Mn5G can be attributed to its relatively uniform and smoother morphology, as well as its more homogeneous amorphous structure. The SEM results show fewer surface protrusions and a lower degree of structural roughness, leading to reduced exposure of active sites and limited electrode–electrolyte interaction. Meanwhile, the XRD pattern suggests a more uniform but less defective structure, which may restrict ion accessibility compared to more disordered systems. Furthermore, at an equimolar Co/Mn ratio, neither the high conductivity advantage of Co-rich systems nor the high surface reactivity of Mn-rich systems is fully realized, resulting in suboptimal charge storage performance. This trend indicates that both Mn-rich and Co-rich systems provide more effective redox-active sites than the intermediate compositions.

**Table 1:** Electrochemical Parameters of Co8Mn2G, Co6Mn4G, Co5Mn5G, Co4Mn6G, and Co2Mn8G Electrodes^a^

active material	*C*_F_ (F/g) (CV)	capacity (C/g) (CV)	*C*_F_ (F/g) (GCD)	capacity (C/g) (GCD)	*R*_s_ (Ω)	*R*_ct_ (Ω)
Co8Mn2G	1268.7	888.1	206.0	103.0	0.68	1.30
Co6Mn4G	1163.0	814.1	174.0	87.0	0.92	0.52
Co5Mn5G	649.7	454.8	100.1	50.1	1.00	1.18
Co4Mn6G	862.2	603.5	156.0	78.0	0.79	1.50
Co2Mn8G	1333.6	933.5	219.7	109.9	0.67	0.44

a CV and GCD curves are respectively measured at 20 mV/s and 1 A/g.

The GCD curves in Figure 2e further corroborate the CV results, where all samples display distinct voltage plateaus rather than linear triangular shapes, confirming their battery-type charge storage behavior. The discharge plateaus are observed in a potential range consistent with the redox peak positions identified in the CV curves, indicating good agreement between the two electrochemical techniques. The specific capacitance and specific capacity are derived from the GCD curves using eq 4 as follows.^41^

4 $$C_{F}=\frac{I\times \Delta t}{\Delta V}$$

where *I* is the applied current density (A/g), and Δ*t* denotes the discharge duration (s). Table 1 shows the specific capacitance and specific capacity of CoMnG calculated using the GCD curves at 1 A/g. Among the samples, Co2Mn8G exhibits the longest discharge duration, corresponding to the highest specific capacitance of 219.7 F/g, corresponding to the specific capacity of 109.9 C/g, followed by Co8Mn2G (206.0 F/g and 103.0 C/g). In contrast, Co5Mn5G again shows the shortest discharge time and lowest capacitance (100.1 F/g, 50.1 C/g), confirming its inferior charge storage capability. In addition, a noticeable IR drop is observed at the beginning of each discharge curve. The Co8Mn2G electrode exhibits the smallest IR drop, indicating lower internal resistance and more efficient electron transport pathways, whereas Mn-rich samples such as Co2Mn8G show a slightly larger IR drop, suggesting increased resistance due to structural disorder and reduced electrical conductivity. The intermediate compositions display moderate IR drops, consistent with their relatively balanced but less optimized structures. The different potential windows used for the CV and GCD measurements are attributed to their different purposes. The CV measurements were performed over a wider potential range (−0.2 to 0.5 V) to identify the electrochemically stable operating window and investigate the redox behavior of the electrode. Based on the CV results, the GCD measurements were subsequently conducted within the practical charge/discharge potential range (0 to 0.5 V). The negative potential region was excluded during the GCD measurements because it does not contribute to the practical charge storage process and may introduce undesirable polarization or side reactions, thereby ensuring a more reliable evaluation of the charge storage performance.

In addition, the Nyquist plots provide further insight into the charge transport characteristics, as shown in Figure 2f. The intercept at the high-frequency region represents the solution resistance (*R*_s_), which is associated with the intrinsic resistance of the electrolyte, electrode material, and current collector interface. The semicircle in the high-frequency region corresponds to the charge-transfer resistance (*R*_ct_), reflecting the kinetics of Faradaic reactions at the electrode/electrolyte interface. The observed variation in *R*_s_ is attributed to the combined effects of intrinsic electronic conductivity, electrode/electrolyte interfacial resistance, and electrolyte accessibility arising from composition-dependent structural evolution, rather than solely the bulk conductivity of the active material. It is observed that *R*_s_ remains relatively low for all samples but shows slight variation with composition, with Co2Mn8G and Co8Mn2G exhibiting lower *R*_s_ values (0.67 and 0.68 Ω, respectively), suggesting better electrolyte accessibility and interfacial contact. In contrast, Co5Mn5G shows a slightly higher *R*_s_ (1.00 Ω), indicating less efficient ion transport. More significantly, the *R*_ct_ value strongly depends on the Co/Mn ratio. Co2Mn8G exhibits the lowest *R*_ct_ (0.44 Ω), indicating the fastest charge transfer kinetics, while the higher *R*_ct_ values for Co4Mn6G and Co2Mn8G suggest slower electron transfer processes. The relatively high *R*_ct_ of Co5Mn5G (1.18 Ω) is consistent with its poor electrochemical performance observed in CV and GCD measurements. The composition-dependent electrochemical behavior can be well understood by correlating it with the structural characteristics revealed by SEM and XRD analyses. The XRD patterns indicate that all samples possess predominantly amorphous structures; however, the intermediate compositions exhibit more uniform and smoother scattering profiles, implying a relatively homogeneous amorphous network. In contrast, both Co-rich and Mn-rich samples show increased structural disorder, consistent with the broadened and fluctuating diffraction features. SEM observations further reveal that Co8Mn2G forms well-defined microspheres with smooth surfaces, which facilitate efficient electron transport and result in low *R*_ct_ and minimal IR drop. On the other hand, Co2Mn8G exhibits highly roughened and nanoparticle-assembled structures with abundant nanoscale protrusions, which significantly increase the specific surface area and provide a large number of electrochemically active sites, thereby enhancing capacitance and capacity despite a slightly higher resistance. In comparison, the intermediate compositions such as Co5Mn5G possess relatively smoother surfaces and more uniform structures with fewer exposed active sites, leading to limited redox activity and inferior electrochemical performance. These results suggest that the electrochemical properties of CoMnG are governed by a balance between electrical conductivity and surface reactivity. Co-rich systems favor fast electron transport and low resistance, while Mn-rich systems maximize the density of active sites and redox participation. Consequently, both ends of the compositional spectrum exhibit higher capacitance and capacity, whereas intermediate ratios suffer from reduced active site availability and suboptimal charge transfer characteristics.

### 3.2 Material and Electrochemical Analysis of Monometallic and Bimetallic Glycerates

To elucidate the effects of metal species and composition, we systematically investigated the structural and electrochemical properties of monometallic and bimetallic glycerates. The morphology of CoG, Co2Mn8G, and MnG was first examined by SEM images, as shown in Figure 3a,d,b,e,c,f. CoG exhibits well-defined and uniform microspheres with smooth surfaces and a narrow size distribution, indicating a typical homogeneous nucleation and isotropic growth process. Such dense and compact structures generally limit the exposure of active sites and the electrolyte accessibility. In contrast, MnG shows irregular, bulk-like particles with severe aggregation and poorly defined morphology. This disordered structure suggests a less controlled nucleation process, likely due to the relatively weaker coordination interaction between Mn^2+^ ions and glycerol molecules, resulting in random particle growth and structural instability. Notably, optimized bimetallic Co2Mn8G presents a different morphology. Although a spherical framework is still partially retained, the surface becomes significantly roughened and is composed of loosely packed nanoscale primary particles. This hierarchical and porous structure is markedly different from that of the smooth CoG spheres and the dense MnG aggregates. The formation of such a structure can be attributed to the synergistic effect between Co^2+^ and Mn^2+^ ions, where the introduction of Mn disrupts the regular crystal growth of cobalt glycerate, inducing heterogeneous nucleation and leading to the assembly of secondary microspheres from the nanoscale building blocks. This structural modulation is expected to provide several advantages, including an increased specific surface area, shortened ion diffusion pathways, and enhanced electrolyte penetration, all of which are beneficial for electrochemical reactions.

**Figure 3:**
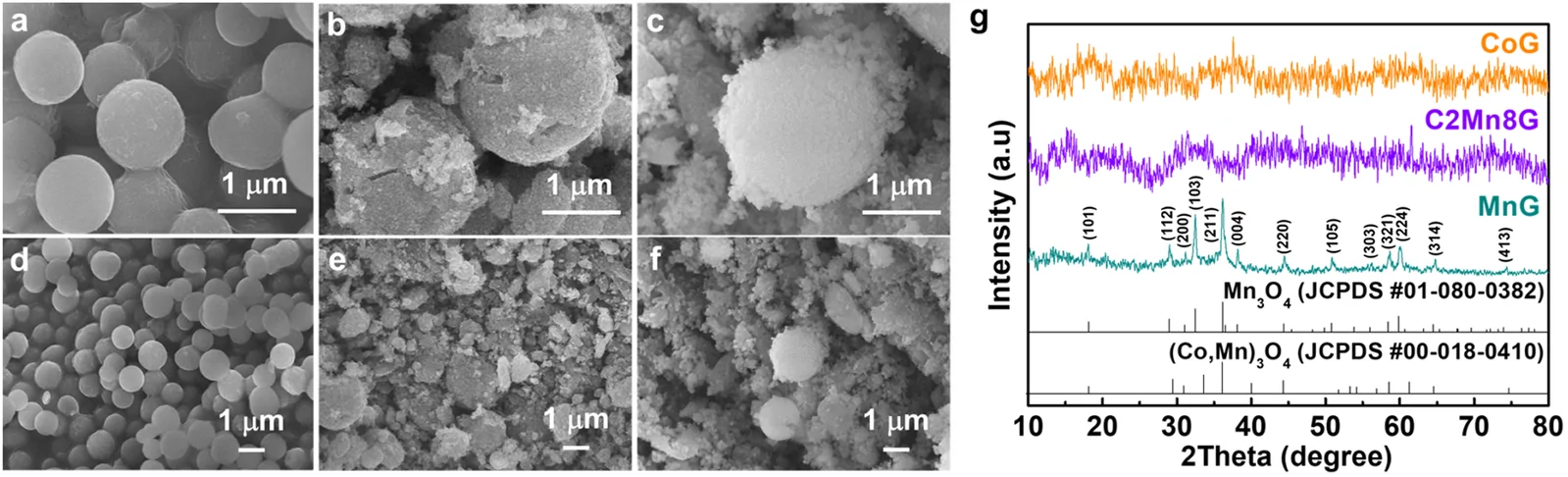
SEM images of (a, d) CoG, (b, e) Co2Mn8G, and (c, f) MnG; (g) XRD patterns of CoG, Co2Mn8G, and MnG.

The crystallographic properties were further examined by XRD patterns shown in Figure 3g. The standard patterns of Mn_3_O_4_ (JCPDS no. 01–080–0382) and (Co,Mn)_3_O_4_ (JCPDS no. 00–018–0410) are shown in this figure for comparison. MnG exhibits multiple sharp and intense diffraction peaks indexed to the spinel Mn_3_O_4_ phase, indicating its high crystallinity and well-ordered structure. This may be attributed to partial oxidation or phase transformation during the solvothermal process or a subsequent drying treatment. In contrast, CoG shows relatively broadened diffraction features with lower intensity, suggesting lower crystallinity and a more disordered framework. For the bimetallic Co2Mn8G sample, the diffraction pattern is highly similar to that of CoG rather than MnG, indicating that the overall crystal structure is primarily governed by the Co-based glycerate framework. The characteristic diffraction peaks of MnG are not clearly observed in Co2Mn8G, suggesting that the Mn species do not form a separate crystalline phase. The Mn incorporation is therefore considered to play a critical role in modulating the crystal structure by introducing defects and disorder into the Co-based framework, which is expected to enhance the ion transport and electrochemical activity. This suggests that Mn ions are likely incorporated into the Co-based glycerate lattice through partial substitution or coordination interaction rather than forming an independent crystalline phase.

Interestingly, a discrepancy between the morphology and crystal structure is observed for the Co2Mn8G sample. As revealed by SEM, the morphology of Co2Mn8G is more similar to that of MnG, exhibiting rough and aggregated nanoscale subunits. However, its XRD pattern closely resembles that of CoG, indicating that the crystal structure is dominated by a Co-based framework. This phenomenon suggests a decoupling between morphological evolution and crystallographic structure in the bimetallic system. The formation of the morphology is primarily governed by the nucleation and growth kinetics during the solvothermal process, where Mn^2+^ ions with relatively weaker coordination ability tend to induce rapid nucleation and disordered aggregation, leading to a rough and porous architecture. In contrast, Co^2+^ ions exhibit stronger coordination interactions with glycerol ligands, favoring the formation of a more stable and dominant crystal framework. Such morphology-structure decoupling is frequently observed in bimetallic coordination systems, where different metal ions play distinct roles in the growth kinetics and lattice stabilization.

XPS was then employed to investigate the surface chemical states and elemental composition of the as-prepared samples. The survey spectra of CoG, MnG, and Co2Mn8G are shown in Figure 4a–c. The survey spectrum of CoG confirms the presence of Co, O, and C elements, while MnG exhibits characteristic signals of Mn, O, and C. In contrast, the Co2Mn8G sample shows the coexistence of Co and Mn signals along with O and C, indicating the successful incorporation of both metal species into a single framework without detectable impurities. These results verify the successful formation of glycerate-based materials. Detailed high-resolution XPS analysis was subsequently performed to further investigate the chemical states of each element. The high-resolution Co 2p spectrum of CoG (Figure 4d) displays two main peaks corresponding to Co 2p_3/2_ and Co 2p_1/2,_ accompanied by characteristic satellite peaks. The coexistence of Co^2+^ and Co^3+^ oxidation states is identified from the deconvoluted peaks. This mixed-valence feature suggests the presence of multiple redox-active centers, which is typically associated with enhanced electron transfer capability. Moreover, the coexistence of Co^2+^/Co^3+^ can facilitate reversible redox transitions, thereby improving the electrochemical reactivity of the material. The pronounced satellite peaks further confirm the partially filled 3d electronic configuration of cobalt species, indicating strong electron correlation effects within the glycerate framework. Similarly, the Mn 2p spectrum of MnG (Figure 4e) reveals the presence of Mn^3+^ and Mn^4+^ species, as evidenced by the deconvoluted Mn 2p_3/2_ and Mn 2p_1/2_ peaks. The coexistence of Mn^3+^/Mn^4+^ can be attributed to partial oxidation and the coordination interaction between manganese ions and glycerate ligands during the solvothermal process. Such mixed-valence states provide multiple redox-active sites and facilitate electron hopping between different oxidation states, thereby improving charge transfer kinetics. For the bimetallic Co2Mn8G sample (Figure 4f,g), the Co 2p spectrum shows a slight shift toward lower binding energy compared to that of CoG, indicating an increase in electron density around Co species. This suggests that electron transfer may occur from Mn to Co, leading to partial electron enrichment of Co sites. Such electronic modulation can facilitate redox reactions by improving the electron-donating capability of cobalt centers. In contrast, the Mn 2p spectrum of Co2Mn8G exhibits nearly identical binding energy positions to those of MnG, indicating that the oxidation state of Mn remains largely unchanged upon bimetallic incorporation. However, a noticeable decrease in peak intensity, along with increased spectral noise, is observed. Considering that XPS is a surface-sensitive technique, this phenomenon suggests a relatively lower surface concentration of Mn species in the bimetallic sample. This implies that Mn may be more embedded within the bulk or partially covered by Co-containing species, resulting in reduced surface exposure. Such a spatial distribution of Co and Mn can be beneficial for electrochemical applications, where surface-exposed Co sites provide active centers for redox reactions, while Mn species contribute to structural stability and additional redox support within the bulk. The combined effect leads to an optimized balance between activity and stability in the bimetallic system. Furthermore, the O 1s spectra (Figure 4h–j) can be deconvoluted into three main components. From higher to lower binding energy, these peaks are assigned to surface hydroxyl groups or adsorbed oxygen species (−OH), defect-related oxygen species associated with oxygen vacancies, and lattice oxygen (M–O), respectively. It is observed that MnG exhibits a significantly higher proportion of hydroxyl groups compared to CoG and Co2Mn8G, indicating a hydroxyl-rich surface that may enhance interfacial wettability and facilitate electrolyte accessibility. In contrast, the Co2Mn8G sample shows a higher proportion of defect-related oxygen and lattice oxygen species than the monometallic counterparts, suggesting a higher concentration of defect-related oxygen species and oxygen vacancies, qualitatively indicating a defect-rich structure. The defect-rich nature is inferred from the combined XRD and XPS analyses, while quantitative determination of defect concentration and spatial distribution requires advanced characterization beyond the scope of this work. Notably, the O 1s peaks of Co2Mn8G shift toward higher binding energy compared to those of CoG and MnG, indicating a decrease in electron density around oxygen species. This shift can be attributed to the electronic interaction between Co and Mn, which modifies the local coordination environment and redistributes electron density within the metal–oxygen bonds. Such electronic modulation further supports the formation of a more polarized M–O structure, which is favorable for enhancing surface reactivity and facilitating charge transfer processes. Overall, the XPS results demonstrate that the bimetallic Co2Mn8G possesses a more complex electronic structure, enriched defect sites, and stronger interfacial interactions than its monometallic counterparts. These features are expected to contribute synergistically to improved electrochemical behavior.

**Figure 4:**
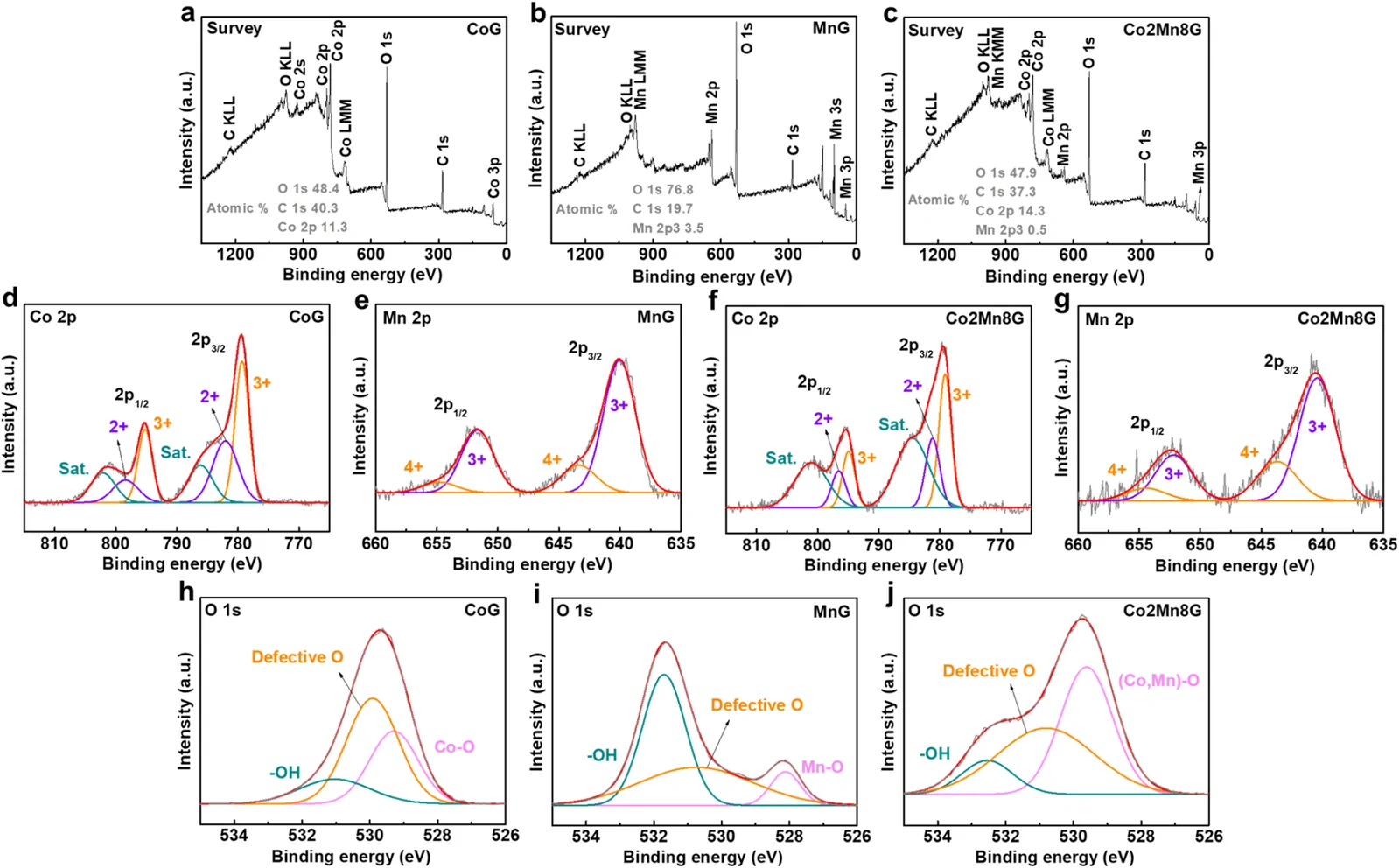
Survey spectra of (a) CoG, (b) MnG, and (c) Co2Mn8G; (d) Co 2p spectrum of CoG, (e) Mn 2p spectrum of MnG, (f) Co 2p, and (g) Mn 2p spectra of Co2Mn8G; O 1s spectra of (h) CoG, (i) MnG, and (j) Co2Mn8G.

The electrochemical performance of CoG, MnG, and Co2Mn8G electrodes was then systematically evaluated by CV, GCD, and Nyquist plots. Figure 5a presents the CV curves of CoG, MnG, and Co2Mn8G electrodes. Notably, Co2Mn8G exhibits a CV profile highly similar to that of MnG, where a quasi-rectangular shape is maintained in the low-potential region, followed by a pair of pronounced redox peaks emerging at higher potentials. This behavior suggests that capacitive contribution dominates at low potential, while Faradaic reactions become significant at higher potential. The single pair of redox peaks observed in MnG can be assigned to the Mn^3+^/Mn^4+^ transition, which can be described by the reaction of MnOOH + OH^–^ ⇌ MnO_2_ + H_2_O + e^–^. For Co2Mn8G, a similar peak feature is observed, but with a broadened peak shape and a slight potential shift. This phenomenon suggests the overlap of Mn-based redox with Co-related reactions. The redox process can therefore be described as a coupled contribution from both metal centers as follows. Co­(OH)_2_ + OH^–^ ⇌ CoOOH + H_2_O + e^–^ and MnOOH + OH^–^ ⇌ MnO_2_ + H_2_O + e^–^. The overlap of these reactions leads to a merged and broadened peak, consistent with the observed CV profile. The quasi-rectangular region at lower potential further indicates enhanced surface-controlled capacitive behavior, which can be attributed to the defect-rich and porous structure identified from structural characterization. In contrast, CoG shows a markedly different response characterized by three distinct pairs of redox peaks distributed over the entire potential window, indicating more complex multistep cobalt redox transitions associated with sequential oxidation processes. These processes are typically associated with sequential oxidation reactions, which can be expressed as Co­(OH)_2_ + OH^–^ ⇌ CoOOH + H_2_O + e^–^ and CoOOH + OH^–^ ⇌ CoO_2_ + H_2_O + e^–^. The presence of multiple redox steps results in separated peak pairs, reflecting distinct reaction pathways and less overlap compared to the bimetallic system. This also indicates that the redox process in CoG is more discretized and less synergistic. Table 2 shows the C_F_ values and specific capacity of CoG, Co2Mn8G, and MnG calculated using the CV curves at 20 mV/s. The *C*_F_ values of 1204.1, 1333.6, and 1309.0 F/g, respectively, corresponding to the specific capacities of 842.9, 933.5, and 916.3 C/g, are obtained for CoG, Co2Mn8G, and MnG electrodes. Notably, Co2Mn8G delivers the highest values among all samples, indicating a more efficient charge storage capability. This improvement can be attributed to the synergistic effect between Co and Mn species, which provides multiple redox-active centers and facilitates more effective Faradaic reactions. In addition, the broadened redox peaks observed in the CV curves of Co2Mn8G suggest a wider potential range for redox transitions, leading to increased charge contribution. Compared to MnG, although a similar CV profile is observed, the enhanced capacity of Co2Mn8G implies improved utilization of active sites, which is likely associated with the optimized electronic structure and defect-rich framework induced by bimetallic incorporation. Meanwhile, the lower values obtained for CoG can be correlated with its more discrete multistep redox behavior and relatively limited accessible surface, as evidenced by its morphology and electrochemical response.

**Figure 5:**
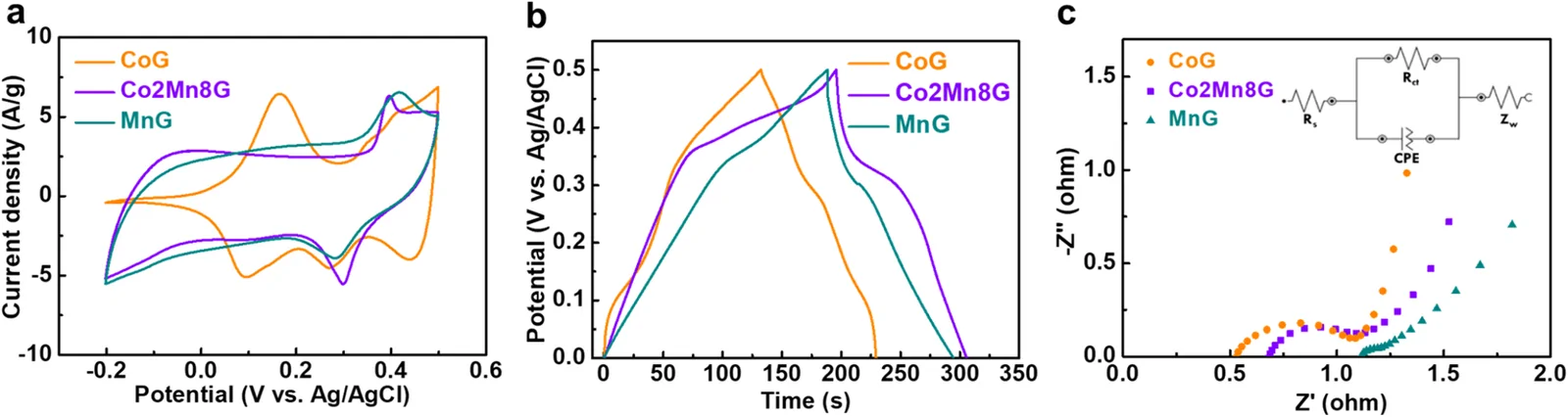
(a) CV curves, (b) GCD curves, (c) Nyquist plots, and the corresponding equivalent circuit of CoG, Co2Mn8G, and MnG electrodes.

**Table 2:** Electrochemical Parameters of CoG, Co2Mn8G, and MnG Electrodes^a^

active material	*C*_F_ (F/g) (CV)	capacity (C/g) (CV)	*C*_F_ (F/g) (GCD)	capacity (C/g) (GCD)	*R*_s_ (Ω)	*R*_ct_ (Ω)
CoG	1204.1	842.9	193.8	96.9	0.51	0.71
Co2Mn8G	1333.6	933.5	219.7	109.9	0.67	0.44
MnG	1309.0	916.3	211.9	106.0	1.12	0.25

a CV curves are measured at 20 mV/s. GCD curves are measured at 1 A/g.

The GCD curves in Figure 5b further support the Faradaic charge storage behavior, where all electrodes exhibit distinct voltage plateaus rather than ideal linear profiles. Co2Mn8G and MnG display similar charge/discharge characteristics, consisting of a relatively linear slope in the low-potential region followed by a pronounced plateau at higher potentials, corresponding to the redox reactions discussed in the CV analysis. In contrast, CoG shows multiple plateau regions, consistent with its multistep cobalt redox transitions. Table 2 shows the *C*_F_ values and specific capacity of CoG, Co2Mn8G, and MnG calculated using the GCD curves at 1 A/g. The C_F_ values of 193.8, 219.7, and 211.9 F/g, respectively, corresponding to the specific capacities of 96.9, 109.9, and 106.0 C/g, are obtained for CoG, Co2Mn8G, and MnG electrodes. A similar trend to that derived from CV is observed, where Co2Mn8G delivers the highest capacitance. This result further confirms the enhanced charge storage capability of the bimetallic system, which can be attributed to the synergistic redox contribution and improved utilization of active sites. In addition, an obvious potential drop at the beginning of the discharge process can be observed for all samples, corresponding to the internal resistance. The IR drop of Co2Mn8G is slightly higher than that of CoG but significantly lower than MnG, indicating that the incorporation of Co effectively reduces the overall internal resistance compared to MnG while maintaining efficient charge transfer. The moderate IR drop observed for Co2Mn8G suggests a balanced electronic conductivity and ion transport capability, which is beneficial for maintaining stable charge/discharge behavior under practical operating conditions. Furthermore, the smaller IR drop of Co2Mn8G compared to MnG implies reduced ohmic loss and improved electrode/electrolyte interface contact, which facilitates rapid electron and ion transport during the charge–discharge process. Combined with the extended discharge time and broader plateau region, these features confirm the enhanced electrochemical utilization of active materials in the bimetallic system.

The charge transfer and ion diffusion behaviors of CoG, MnG, and Co2Mn8G electrodes were further investigated by EIS, as shown in the Nyquist plots (Figure 5c). All samples exhibit a typical impedance response consisting of a semicircle in the high-frequency region and a sloped straight line in the low-frequency region. The extracted *R*_s_ values are 0.51, 0.67, and 1.12 Ω for CoG, Co2Mn8G, and MnG, respectively. The relatively low *R*_s_ value of CoG indicates superior intrinsic conductivity, while the highest *R*_s_ value for MnG suggests sluggish electron transport. On the other hand, MnG exhibits the smallest *R*_ct_ value of 0.25 Ω, indicating the fastest interfacial charge transfer kinetics. However, this advantage is offset by its large *R*_s_ value and structural instability. CoG shows a larger *R*_ct_ value of 0.71 Ω, suggesting slower charge transfer due to its relatively compact and less accessible structure. Notably, Co2Mn8G exhibits an intermediate *R*_ct_ value of 0.44 Ω, which is significantly lower than that of CoG but slightly higher than that of MnG. This indicates that the incorporation of Mn effectively reduces the charge transfer resistance of the Co-based system, while maintaining a more stable conductive framework than MnG. The balanced *R*_s_ and *R*_ct_ values of Co2Mn8G suggest a synergistic optimization of both bulk conductivity and interfacial charge transfer. In the low-frequency region, the inclined line is associated with the Warburg impedance, which reflects ion diffusion behavior within the electrode. Ideally, a more vertical line indicates lower diffusion resistance and faster ion transport. MnG with the steepest slope suggests relatively fast ion diffusion, whereas CoG with a more gradual slope indicates more pronounced diffusion limitation. Importantly, Co2Mn8G displays an intermediate slope between MnG and CoG, implying moderated ion diffusion behavior. This suggests that while the diffusion rate in Co2Mn8G is not the fastest, it is significantly improved compared to CoG and is more structurally stable than MnG. The hierarchical porous architecture and defect-rich framework of Co2Mn8G facilitate electrolyte penetration and shorten ion diffusion pathways, while avoiding the excessive structural disorder observed in MnG. Overall, although MnG exhibits the lowest charge transfer resistance and fastest apparent ion diffusion, its high internal resistance and poor structural integrity limit its practical electrochemical performance. In contrast, CoG suffers from sluggish charge transfer and ion diffusion due to its dense structure. The bimetallic Co2Mn8G achieves a well-balanced electrochemical kinetic profile, featuring moderate *R*_s_, reduced *R*_ct_, and improved ion diffusion behavior. This balanced optimization, together with the synergistic electronic interaction between Co and Mn species, contributes to enhanced charge storage capability and superior overall electrochemical performance compared to the monometallic counterparts. Although direct conductivity measurements were not performed, the relatively low *R*_s_ and *R*_ct_ values obtained from the Nyquist plots indicate improved electron transport characteristics.

Figure 6 presents the rate performance of CoG, Co2Mn8G, and MnG electrodes, with CV and GCD measurements at various scan rates and current densities used to elucidate their charge storage behavior. The CV curves at different scan rates (Figure 6a–c) reveal pronounced redox features for all electrodes, confirming dominant Faradaic charge storage behavior. CoG exhibits multiple pairs of redox peaks, corresponding to stepwise Co^2+^/Co^3+^ and Co^3+^/Co^4+^ transitions, indicating complex multistep reactions. In contrast, MnG shows relatively simpler redox behavior dominated by the Mn^3+^/Mn^4+^ couple, along with a quasi-rectangular background at lower potentials, suggesting a mixed capacitive-Faradaic contribution. Notably, Co2Mn8G displays broadened and overlapped redox peaks, integrating characteristics of both CoG and MnG. This feature reflects synergistic coupling between Co and Mn redox centers, which facilitates more continuous electron transfer and enhances reaction reversibility. With increasing scan rate, the current response increases while the CV profiles remain well maintained, indicating good structural stability and favorable charge transport kinetics. Slight polarization at higher scan rates is observed for all electrodes, which can be attributed to diffusion limitations under rapid charge/discharge conditions. The GCD curves (Figure 6d–f) further confirm the pseudocapacitive behavior, exhibiting nonlinear profiles with distinct voltage plateaus. CoG shows multiple discharge plateaus associated with its multistep cobalt redox reactions, whereas MnG presents a more symmetric and smooth profile, indicating relatively uniform redox activity. Co2Mn8G exhibits broadened plateau regions combined with sloping characteristics, suggesting the coexistence and overlap of Co- and Mn-related redox processes. Moreover, the longer discharge time of Co2Mn8G compared to the monometallic electrodes indicates higher capacitance and more efficient utilization of electroactive sites.

**Figure 6:**
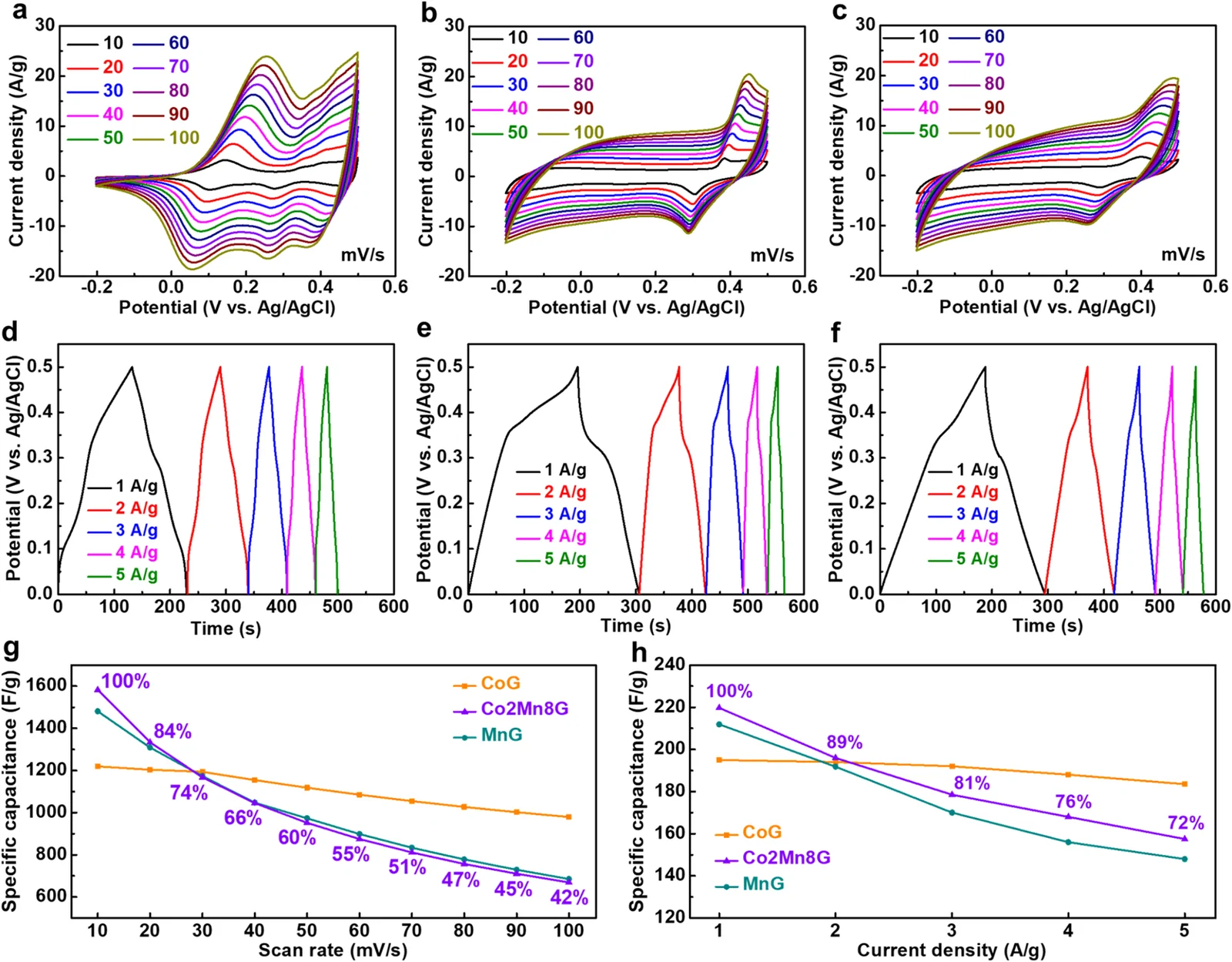
CV curves at different scan rates for (a) CoG, (b) Co2Mn8G, and (c) MnG electrodes; GCD curves at different current densities for (d) CoG, (e) Co2Mn8G, and (f) MnG electrodes; relationships of specific capacitances to (g) scan rates and (h) current densities of CoG, Co2Mn8G, and MnG.

The rate performance is summarized in Figure 6g,h. CoG exhibits the highest capacitance retention, indicating superior rate capability, while Co2Mn8G shows intermediate performance but consistently outperforms MnG. For Co2Mn8G, the capacitance retention decreases from 100% to 84%, 74%, 66%, 60%, 55%, 51%, 47%, 45%, and 42% as the scan rate increases from 10 to 100 mV/s. Under increasing current density, it retains 80%, 81%, 76%, and 72% of its initial capacitance at 2, 3, 4, and 5 A/g, respectively. These results indicate that Co2Mn8G achieves a balanced rate performance between CoG and MnG. Despite its lower retention compared to CoG, Co2Mn8G delivers the highest specific capacitance at low scan rates and current densities. This enhanced capacity originates from the synergistic effect between Co and Mn redox centers. The coexistence of multiple oxidation states (Co^2+^/Co^3+^/Co^4+^ and Mn^3+^/Mn^4+^) provides abundant electroactive sites, enabling more extensive Faradaic reactions. Meanwhile, the incorporation of Mn modifies the electronic structure and promotes charge transfer, improving the utilization efficiency of active materials. Together with the broadened and coupled redox processes observed in CV, these factors result in superior capacitance for Co2Mn8G, despite its slightly reduced rate capability.

The enhanced electrochemical performance of the bimetallic glycerate electrode can be attributed to the synergistic structural and electronic effects induced by the Mn incorporation. As observed from the SEM images, the introduction of Mn disrupts the regular growth of cobalt glycerate, resulting in a rougher surface morphology with an abundance of structural defects. Such a morphology provides a larger electrochemically active surface area and exposes more accessible redox-active sites for Faradaic reactions while also facilitating electrolyte penetration and ion diffusion. In addition, XPS analysis reveals an electronic interaction between Co and Mn, which modifies the local electronic structure and promotes electron transfer during the charge–discharge process. The coexistence of Co and Mn redox centers further offers multiple reversible oxidation states, contributing to an enhanced pseudocapacitive charge storage. Therefore, the combined structural and electronic advantages of the bimetallic glycerate effectively improved the specific capacitance, rate capability, and long-term cycling stability of the optimized electrode.

To further elucidate the charge storage mechanism, the relationship between peak current (*i*) and scan rate (ν) was analyzed according to the power-law equation (*i* = *a*ν^*b*^).^36^ Figure 7a–c respectively present the corresponding log­(*i*) versus log­(ν) plots for CoG, Co2Mn8G, and MnG electrodes at different redox peaks. All peaks are labeled according to the inset figures. Three pairs of redox peaks are clearly resolved for CoG, whereas only a single pair of redox peaks is observed for Co2Mn8G and MnG. The good linearity indicates that the charge storage behavior of all electrodes can be well described by this model. The extracted b-values provide insight into the dominant charge storage mechanism, where a value of 0.5 corresponds to a diffusion-controlled process and a value of 1.0 indicates a surface-controlled capacitive behavior.

**Figure 7:**
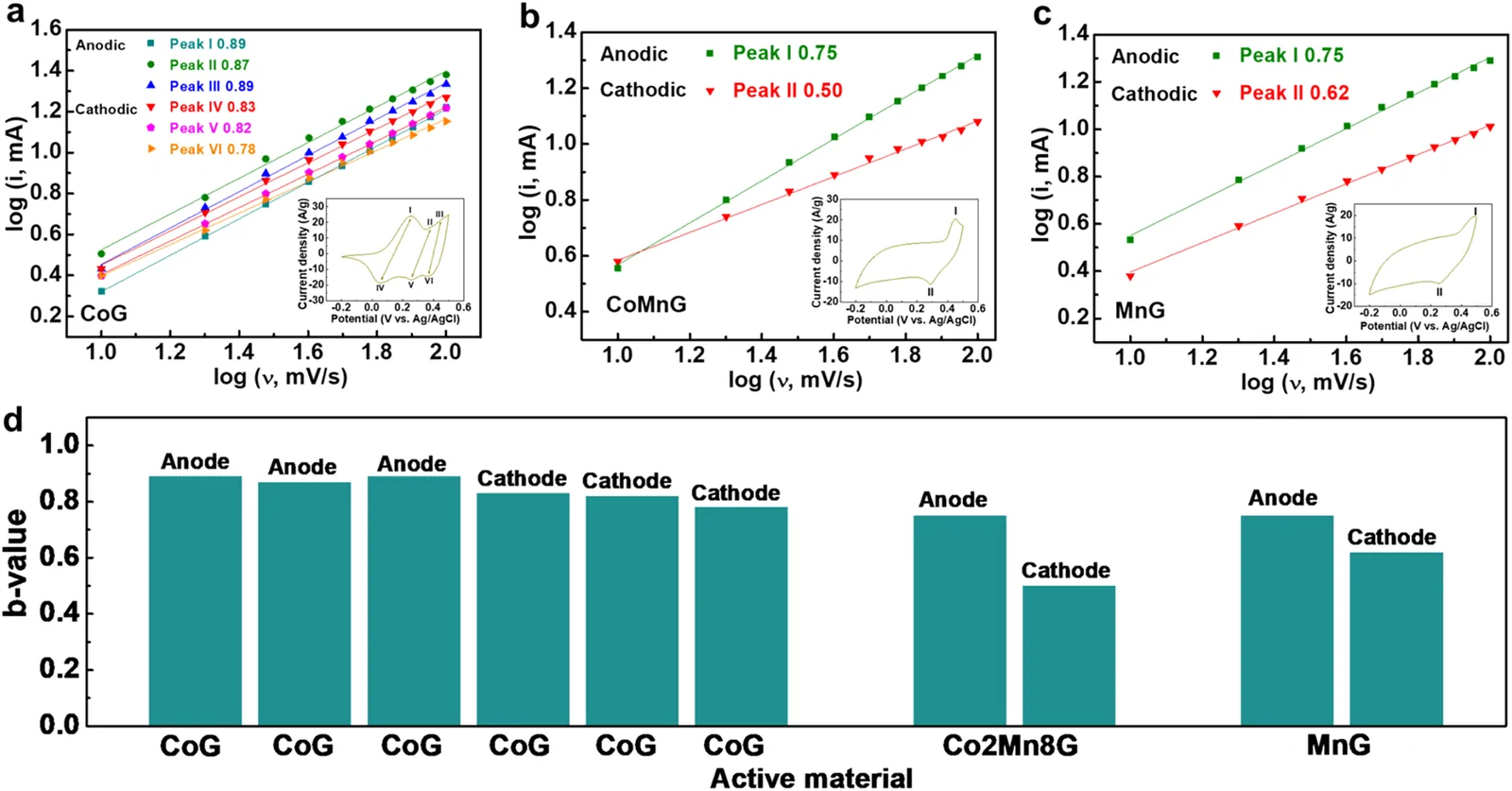
Relationships of log­(*i*) and log (scan rate) for (a) CoG, (b) Co2Mn8G, and (c) MnG electrodes; (d) relationships of *b*-value to the active materials.

As shown in Figure 7d, CoG exhibits relatively high *b*-values (0.78–0.89), suggesting that its charge storage is predominantly governed by surface-controlled capacitive processes. This is consistent with its CV characteristics and implies that the electrochemical reactions mainly occur at or near the electrode surface. However, such behavior typically limits the utilization of bulk active materials, leading to their relatively lower specific capacity. In contrast, MnG shows lower *b*-values (0.62 and 0.75), indicating a stronger diffusion-controlled contribution. This suggests that charge storage is largely governed by ion diffusion into the bulk. Although this mechanism is beneficial for achieving higher capacity, it is often accompanied by sluggish kinetics and higher internal resistance, which is consistent with the EIS results showing a large *R*_s_ value. Notably, Co2Mn8G exhibits *b*-values of 0.50 and 0.75, indicating a more pronounced diffusion-controlled behavior than both CoG and MnG. The b-value of 0.50 suggests a dominant diffusion-limited Faradaic process, while the value of 0.75 reflects a mixed contribution. Compared to CoG, the reduced *b*-values indicate enhanced bulk redox participation and improved utilization of active materials. Compared to MnG, the relatively higher *b*-value at one redox peak suggests improved surface reaction kinetics. This result indicates that Co2Mn8G does not simply exhibit an intermediate mechanism, but rather achieves a diffusion-dominated yet kinetically optimized charge storage process. Such behavior can be attributed to the synergistic effects of the bimetallic system: Mn incorporation promotes ion diffusion by inducing structural disorder and porosity, while Co enhances electrical conductivity and accelerates surface redox reactions. Consequently, Co2Mn8G achieves an effective balance between diffusion-controlled capacity and reaction kinetics, which is consistent with its superior electrochemical performance observed in CV, GCD, and EIS measurements.

### 3.3 Electrochemical Performance Analysis of the Two-Electrode Supercapacitor

The electrochemical performance of the assembled two-electrode supercapacitor was evaluated to determine the appropriate operating voltage and further assess its charge storage characteristics. Figure 8a shows the CV responses measured at 20 mV/s under different potential windows. The inset of Figure 8a shows the CV curve of the rGO negative electrode. Owing to its excellent electrical conductivity, large specific surface area, and predominantly electric double-layer capacitive behavior, rGO serves as an ideal negative electrode in an asymmetric device. It provides rapid electron transport and complements the Faradaic charge storage of the Co2Mn8G positive electrode, thereby expanding the operating voltage window and improving the overall energy density. As the voltage range increases, the enclosed area of the CV curves becomes larger, indicating an increase in the stored charge. However, when the upper voltage exceeds 1.3 V, the curves exhibit noticeable deformation and a sharp current increase at high potentials, suggesting the onset of side reactions that may compromise stability. Therefore, 1.3 V was selected as the optimal operating voltage. The effect of the voltage window on the charge/discharge behavior is further examined by GCD measurements at 1 A/g (Figure 8b). At higher voltages, a more obvious IR drop appears at the beginning of the discharge process, indicating an increased internal resistance. In comparison, the curve at 1.3 V shows a more symmetric and stable profile, implying an improved charge transfer efficiency. The CV curves at different scan rates within 1.3 V are presented in Figure 8c. The general shapes are well-preserved as the scan rate increases, indicating good reversibility of the redox reactions. Meanwhile, the current response increases with the scan rate, reflecting favorable reaction kinetics. Although slight polarization occurs at higher scan rates, the overall stability of the curves suggests that the device can operate reliably under fast-charging and -discharging conditions. Figure 8d shows the GCD curves measured at various current densities. The nearly symmetric charge/discharge profiles confirm the good reversibility of the device. Even at higher current densities, the curve shapes remain largely unchanged, indicating a stable performance under high-rate operation.

**Figure 8:**
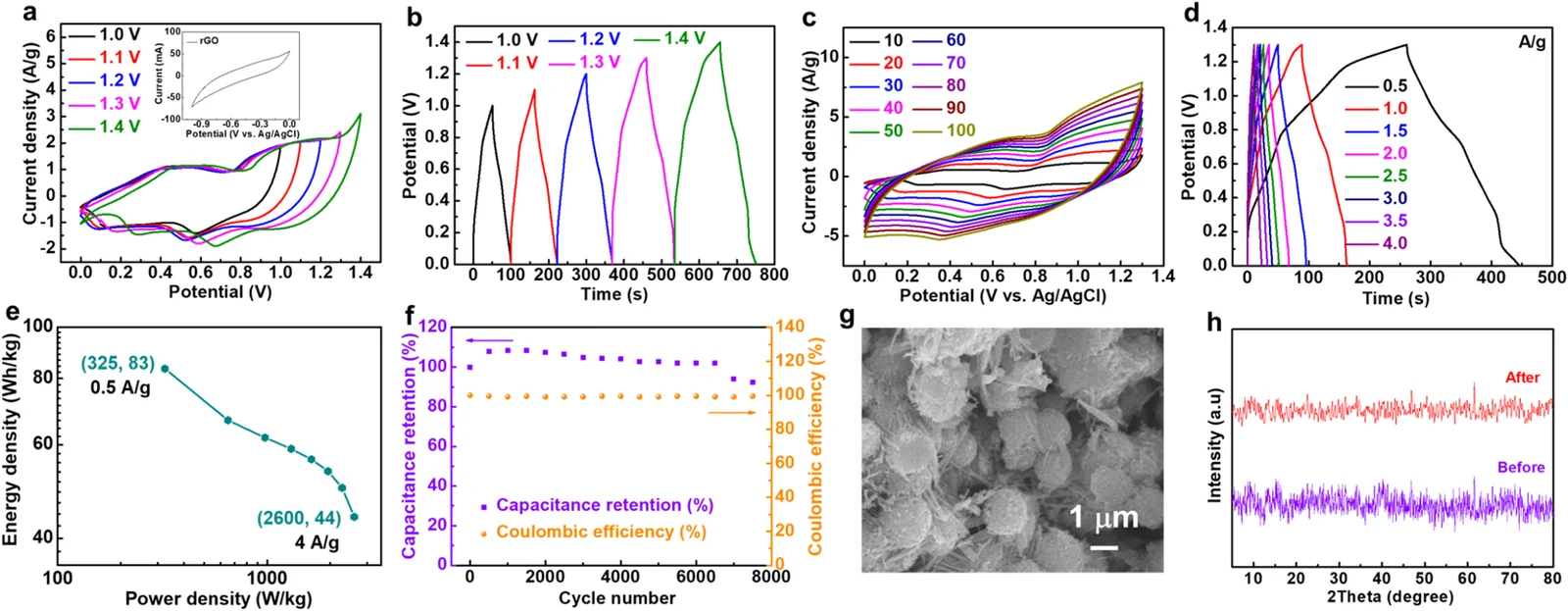
(a) CV curves with different potential windows at 20 mV/s (the inset shows the CV curve of rGO), (b) GCD curves with different potential windows at 1 A/g, (c) CV curves with a potential window of 1.3 V at different scan rates, (d) GCD curves with a potential window of 1.3 V at different current densities, (e) Ragone plot, and (f) relationships of *C*_F_ retentions and Coulombic efficiency to cycle number measured using a current density of 1 A/g in 7500 cycles for the two-electrode supercapacitor composed of a rGO negative electrode and a Co2Mn8G positive electrode. (g) The SEM image after the stability test and (h) XRD patterns before and after the stability test of a Co2Mn8G positive electrode.

The energy and power characteristics of the device are summarized in the Ragone plot (Figure 8e). The energy density decreases with an increase in power density, reflecting the typical trade-off behavior of supercapacitors. A maximum energy density of 83 Wh/kg was obtained at a power density of 325 W/kg. Also, at the maximum power density of 2600 W/kg, the energy density still remained at 44 Wh/kg. The cycling stability of the device is also evaluated at 1 A/g for 7500 cycles, as shown in Figure 8f. After 7500 cycles, the capacitance retention remains at approximately 90%, while the Coulombic efficiency remains close to 100% throughout the test. The stable efficiency indicates a highly reversible charge storage process, while the gradual decrease in the capacitance may be related to minor structural or interfacial changes during long-term cycling. Overall, the device exhibits good electrochemical stability and reliable performance. To further evaluate the structural stability of the Co2Mn8G electrode under alkaline electrochemical conditions, postcycling SEM and XRD analyses were performed after the electrochemical measurements. As shown in Figure 8g, the electrode retains its original morphology without obvious collapse or aggregation after cycling. In addition, the postcycling XRD pattern (Figure 8h) remains essentially identical to that of the pristine sample, with no detectable new diffraction peaks or significant peak shifts. These observations indicate that the bimetallic glycerate framework remains structurally stable during repeated charge/discharge processes, confirming that the excellent cycling performance is closely associated with the preserved crystal structure and morphology.

Table 3 compares the electrochemical performance of the assembled BSH device with recently reported Co- and Mn-based electrode materials. Most previously reported Co/Mn-based systems exhibit energy densities ranging from 29.39 to 65 Wh/kg, with cycling stability between 82.6% and 97% after 5000 to 25,000 charge/discharge cycles. In comparison, the Co2Mn8G-based BSH developed in this work delivers a high specific capacitance of 1333.6 F/g at 20 mV/s (219.7 F/g at 1 A/g), together with an outstanding energy density of 83 Wh/kg at a power density of 325 W/kg. Furthermore, the device maintains 90% capacitance retention with nearly 100% Coulombic efficiency after 7500 cycles, demonstrating excellent reversibility and long-term electrochemical stability. These results indicate that the synergistic effect of the bimetallic Co–Mn glycerate effectively enhances charge storage capability while maintaining excellent cycling durability, making it highly competitive among recently reported Co- and Mn-based BSH systems.

**Table 3:** Partial Lists of Electrochemical Performance of BSHs with Co- and Mn-Based Active Materials

active material	capacity	energy density @power density	*C*_F_ retention & Coulombic efficiency @ cycle number	refs
Mn–Co–Se	509 C/g @ 1 A/g	46.2 Wh/kg @ 800 W/kg	89.2% and 100% @ 5000	^42^
Mn–Co–Se	270 mAh/g @ 2 A/g	29.39 Wh/kg @ 399.9 W/kg	97% and 98% @ 25,000	^43^
Mn–Co–Se	1656 F/g @ 1 A/g	55.1 Wh/kg @ 880 W/kg	90.2% and 97% @ 8000	^44^
Co–Mn–Se	519 C/g @ 1 A/g	35.47 Wh/kg @ 11500 W/kg	82.6% & NA @ 10,000	^45^
Co–Mn–Fe–Se	1112.5 C/g @ 1 A/g	65 Wh/kg @ 802.5 W/kg	91% and 98.1% @ 10,000	^46^
Co2Mn8G	1333.6 F/g @ 20 mV/s, 219.7 F/g @ 1 A/g	83 Wh/kg @ 325 W/kg	90% and 100% @ 7500	this work

## 4 Conclusions

This study demonstrates a strategy for tuning cobalt–manganese glycerate electrodes via compositional regulation to optimize supercapacitor performance. Varying the Co/Mn ratio effectively controls nucleation and growth behavior, leading to a structural evolution from smooth microspheres to rough, nanoparticle-assembled architectures with increased disorder. The Mn-rich Co2Mn8G exhibits the best performance due to its defect-rich and porous structure, which enhances electrolyte accessibility and provides abundant active sites, while Co incorporation improves electrical conductivity and structural stability. As a result, Co2Mn8G shows the highest specific capacitance of 1333.6 F/g at 20 mV/s and 219.7 F/g at 1 A/g, outperforming both Co-rich and intermediate compositions. The assembled device achieves a maximum energy density of 83 Wh/kg at the power density of 325 W/kg and maintains a capacitance retention of 90% and Coulombic efficiency of 100% throughout 7500 cycles. Future work may focus on extending the compositional design to multimetal systems to further improve durability and charge storage performance.
